# Health-related risky behaviors in Chinese adolescents with autism: a cross-sectional study

**DOI:** 10.1186/s13034-021-00390-6

**Published:** 2021-07-30

**Authors:** Yajing Sun, Xue Li, Lingzi Xu, Zenghui Ma, Yulu Yang, Tingni Yin, Zilin Gao, Xiaoyun Gong, Lei Li, Qinyi Liu, Xinzhou Tang, Jing Liu

**Affiliations:** 1 Peking University Institute of Mental Health, Peking University Sixth Hospital, Key Laboratory of Mental Health, National Health Commission (Peking University), National Clinical Research Center for Mental Disorders (Peking University Sixth Hospital), No. 51 Huayuan North Road, Haidian District, Beijing, 100191 China; 2 Children’s and Women’s Hospital, Quanzhou, 362000 China

**Keywords:** Autism, Adolescents, Health-related risky behaviors, Risk factors

## Abstract

**Background:**

Health-related risky behaviors (HRB) generally refer to behaviors that have a negative influence on health and quality of life. HRB in adolescents with autism have not been well understood so far. We aim to explore health-related risky behaviors and their risk factors with autistic adolescents.

**Methods:**

In this study, 150 adolescents with autism and 150 neurotypical adolescents were enrolled. Participants in both groups completed the Adolescent Health-Related Risky Behavior Inventory (AHRBI). Autism Spectrum Screening Questionnaire (ASSQ), Wechsler Intelligence Scale, Theory of Mind (ToM) Test, Zung Self-rating Anxiety Scale (SAS), Zung Self-rating Depression Scale (SDS), and Self-Esteem Scale (SES) were also assessed in the autism group to explore risk factors.

**Results:**

The results showed that the total score of AHRBI and scores of "aggression and violence (AV)", "suicide or self-injury (SS)", "health-compromising behavior (HCB)", and "unprotected sex (US)" subscales in the autism group were significantly higher than those in the control group (Z value = − 4.58 ~ − 2.26, all P < 0.05). Anxiety, depression, low self-esteem, low IQ score, low ToM test score, increasing age, and communication disorder were found as risk factors for health-related risky behaviors in autistic adolescents.

**Conclusions:**

Adolescents with autism have more health-related risky behaviors than neurotypical adolescents. We should pay attention to the emotional state, self-esteem, cognitive function, and verbal communication levels of autistic adolescent with health-related risky behaviors.

**Supplementary Information:**

The online version contains supplementary material available at 10.1186/s13034-021-00390-6.

## Background

Autism spectrum disorder (ASD) refers to a group of neurodevelopmental disorders, including autism, asperger's syndrome (AS), and pervasive developmental disorder-not otherwise specified (PDD-NOS) [1]. As reported in 2020, the prevalence of ASD has increased to almost one in every 54 persons [2]. Belong to ASD, autism is characterized by early-onset impairment in communication, social interaction, and repetitive and stereotyped patterns of behavior [3]. This disorder can seriously impair autistic individuals’ social function and impose a heavy burden on both families and society [4, 5].

Previous studies have found that autism was associated with a variety of health-related risky behaviors (HRB), which may have adverse effects on their health and quality of life [6–8]. These behaviors including aggression and violence, smoking, alcohol and drug addiction, sexual behaviors related to accidental pregnancy or sexually transmitted infections, unhealthy eating habits, and lack of exercise may cause psychosomatic injuries [9]. HRB early appear during adolescence, while may persist into adult life and cause negative consequences in diverse areas, such as impairment of social function and high incidence of chronic diseases [10, 11]. Thus, a great attention should be paid to such behaviors with autism.

By now, aggression is one of the most commonly investigated HRB with ASD. This behavior has high prevalence (49%–68%) in children and adolescents with ASD [12]. Hirota et al. found that irritability and aggression are related to depressed mood and oppositionality [13]. More relevant factors are needed to be explored. Self-injurious behaviors are also common in autistic adolescents. Handen et al. reported that among 302 ASD individuals who aged 4–20 years old and were hospitalized in 6 psychiatric institutions, 74% were rated to have self-injurious behavior by nurses [14]. However, the majority of researchers have concentrated on single behaviors and have not comprehensively described HRB in autistic individuals. Empirical studies have found that various types of dangerous or problematic behaviors were associated with each other and relevant researchers have proposed a theory with inclusion of three main systems: personality, perceived environment, and behavior systems. These systems are combined to influence behavior-associated problems [15, 16]. Therefore, it is highly essential to take the multifaceted nature of risky behaviors into consideration when studying this issue in individuals with autism.

In order to comprehensively explore HRB in adolescents with autism, an efficient evaluation tool is of great importance. The majority of these tools are self-report questionnaires, such as the Youth Risk Behavior Survey (YRBS) questionnaire [17], the Adolescent Risk Inventory (ARI) [18], the Adolescent Risk Behavior Screening Form [19], and the Adolescent Risk Behavior Questionnaire (RBQ-A). However, these questionnaires have some disadvantages: (1) The validity data of YRBS need to be improved; (2) ARI is not able to fully evaluate individuals’ health-related risky behaviors because it only assesses three types of risky behaviors; (3) In ARBS, there are only 9 questions, five of which are related to substance abuse; (4) RBQ-A has not been formally published and a thorough psychometric survey has not been conducted.

To overcome the shortcomings of the above-mentioned questionnaires, Wang et al. [20] developed the Adolescents Health-Related Risky Behavior Inventory (AHRBI), measured six kinds of risky behaviors in adolescents, and confirmed that the questionnaire possessed good reliability and validity. The AHRBI has been used for the assessment of HRB in adolescents with diverse characteristics, such as antisocial personality, periodontitis, or psychosis [21–24]. It was also used in a study that found HRB were increased in adolescents with high-functioning autism, who had more aggressive behavior, unhealthy eating behavior, self-injurious behavior, and breaking discipline than their peers [25]. However, this study enrolled only 50 adolescents with autism and did not explore factors associated with HRB in the autism group.

Therefore, in order to comprehensively explore HRB in a large sample of adolescents with autism, we enrolled 150 autistic adolescents and 150 neurotypical adolescents. We hypothesized that individuals in the autism group might have more HRB than neurotypical adolescents and that these risky behaviors might be correlated with their core symptoms, cognitive function, anxiety or depression, and family situation.

## Methods

### Study design

This study was a cross-sectional study conducted from March 2018 to December 2019. Autism and control groups were included in our study. All participants were required to complete AHRBI and their parents fill the general condition form. Additionally, participants in the autism group were invited to complete SAS, SDS, SES, IQ test, and ToM test, and their parents were invited to fill in ASSQ according to their children's situation. Childhood Autism Rating Scale (CARS) was assessed on the individuals with autism. The parents and participants in both groups were interviewed according to Schedule for Affective Disorders and Schizophrenia for School-Age Child-Present and Lifetime Version (K-SADS-PL) by two psychiatrists. 150 adolescents with autism were in the autism group and 150 neurotypical adolescents were in the control group.

### Participants

The autism group’s inclusion criteria were as follows: (1) Individuals who were diagnosed with autism by two experienced child psychiatrists according to the autism-associated criteria presented in DSM-IV; (2) Total score of Childhood Autism Rating Scale (CARS) evaluated by child psychiatrists was at least 30; (3) Individuals who were able to understand and fill out the AHRBI; (4) Individuals’ age was between 12 and 19 years old. In addition, the autism group’s exclusion criterias were as follows: (1) Individuals with mental disorders who were diagnosed by clinical evaluation and K-SADS-PL (e.g., psychotic disorders, manic disorder or major depressive); (2) Individuals who had a severe medical condition; and (3) Individuals who cannot understand the AHRBI or cannot cooperate in filling it out. There were 150 individuals with autism who were enrolled (median age = 14.5 years old, 124 males, 26 females). The enrolled participants with autism were from the Child and Adolescent Mental Health Department of Peking University Sixth Hospital (Beijing, China).

The participants in the control group were enrolled from a public school and matched to the autism group by gender and age on a 1:1 ratio. The inclusion criteria included: (1) Individuals who aged between 12 and 19 years old; (2) Individuals who had typical development; (3) Individuals who could understand and fill out the AHRBI. The exclusion criteria included: (1) Individuals with mental disorders who were diagnosed by clinical evaluation and K-SADS-PL. We used clinical assessment and diagnosis to exclude autism; (2) Individuals who had a severe medical condition; and (3) Individuals who cannot understand the AHRBI or cannot cooperate in filling it out. The control group consisted of 150 healthy participants (median age = 14.5 years old, 124 males, 26 females).

### Measures

#### Demographic characteristics

The general condition sheets were self-designed by investigators, consisting of a series of items regarding children’s gender, race, status of residence, age, father’s educational level, mother’s educational level, family relationships, family financial situation and family history of psychiatric disorders. Race included Han and other ethnic groups. The status of residence was divided into cities, counties, and rural areas. We defined: 1 = city, 2 = town, 3 = rural area. Father's and mother's educational levels included primary school, middle school, high school, undergraduate, and postgraduate. We defined: 1 = Primary School, 2 = Middle School, 3 = High School, 4 = Undergraduate, 5 = Postgraduate. Family relationships included harmonious, fine, disharmony, parents divorced. We defined: 1 = Harmonious, 2 = Fine, 3 = Disharmony, 4 = Parents Divorced. Family financial situation included five types. Each family was divided into different groups according to their annual income (unit: RMB). We defined: 1 =  < 30000yuan, 2 = 30,000 ~ 50000yuan, 3 = 50,000 ~ 70000yuan, 4 = 70,000 ~ 100000yuan, 5 =  > 100000yuan. Family history of psychiatric disorders included positive and negative outcomes.

##### CARS

The CARS includes 15 items, and each item is rated on a scale of 1 to 4. According to a composite score, participants can be classified as non-autism, mild autism, moderate autism, and severe autism [26]. The total score of the scale ranged from 15 to 60, and we classified a participant enrolled in the autism group should get a score higher than 30 [27].

##### K-SADS-PL

K-SADS-PL is a semi-structured diagnostic test, which is used to evaluate psychopathological disorders in children and adolescents (age 6–18) [28]. K-SADS-PL was herein performed as an enrollment to exclude individuals in both groups who had comorbid diagnosis of other severe mental disorders (e.g., psychotic disorders, major depression or manic disorder) evaluated by psychiatrists.

##### AHRBI

The AHRBI includes 38 items related to HRB [20]. All items in this scale were shown in Additional file 1: Table S1. In the current study, the participants were asked to indicate how often they experienced each symptom in the period of one year on a 5-point Likert-like scale (1 = never, 2 = rarely, 3 = sometimes, 4 = often and 5 = very often). The inventory has six subscales: “aggression and violence (AV)”, “health-compromising behavior (HCB)”, “rule breaking (RB)”, “unprotected sex (US)”, “suicide and self-injury (SS)”, and “substance use (SU)”. The higher the factor score, the more serious the health-related risky behavior. The higher the total score of the scale, the more the individual’s overall health-related risky behaviors. The total score of AHRBI ranged from 38 to 190.

##### SDS

The SDS contains 20 items, and we used it to assess the symptoms of depression in participants in the autism group. Each item was rated with respect to how participants felt using a four-point Likert-type scale. Options included: 1 = little or no time, 2 = a small part of the time, 3 = quite a lot of time, 4 = most or all of the time. Forward scoring questions were scored as 1, 2, 3, and 4; reverse scoring questions were scored as 4, 3, 2, and 1. Reverse scoring question number: 2, 5, 6, 11, 12, 14, 16, 17, 18, 20. The raw total score of the SDS ranged from 20 to 80, while the results were typically presented as the SDS index, which was obtained by expressing the total score converted to a scale of 100 points [29].

##### SAS

The SAS is a self-report scale whose 20 items cover a series of anxiety symptoms [30]. In the current research, participants were instructed to choose how often they experienced each symptom over the last week given on a four-point Likert-type scale, ranging from 1 (none) to 4 (most). Items includes both positive and negative experiences. Forward scoring questions were scored as 1, 2, 3, and 4; reverse scoring questions were scored as 4, 3, 2, and 1. Reverse scoring question number: 5, 9, 13, 17, 19. The standard total score of the SAS ranges from 25 to 100. The higher the standard score, the more serious the symptom.

##### SES

The SES contains 10 items, and we used it to assess the self-respect and self-acceptance of participants in the autism group. They were asked to rate on a four-point Likert-type scale, ranging from 1 for totally disagree to 4 for totally agree. Items 1, 2, 4, 6, 7, 8 were worded in positive terms, while items 3, 5, 9,10 were worded in negative terms [31]. The total score range is 10–40. The higher the score, the higher the self-esteem.

#### ToM

Theory of mind refers to the individual's ability to understand self and others' intentions or beliefs and to explain or predict the behavior of others. We used two false belief tasks as a test of theory of mind: the first-order false belief task and second-order false belief task. The first-order false belief task and second-order false belief task were previously described by Baron-Cohen [32] and Sullican [33], respectively. First-order false belief task refers to the ability to understand the false belief of others. Second-order false belief task refers to the inference or cognition of others' beliefs about another person, that is, the recursive thinking of others' psychological activities. Both tests had two kinds of questions, control question and test question. Control questions answered incorrectly, scored " 0 "; only control questions answered correctly, scored " 1 "; both control and test questions answered correctly, scored " 2 ".

##### ASSQ

The ASSQ has good properties for screening the broader phenotype of autistic traits. It consists of 27 items rated on a three-point scale, ranging from 0 (normal) to 2 (definite abnormality) [34]. There were three subscales within the ASSQ, including social interaction disorder, communication barriers, and limited and repetitive behaviour. The total score ranged from 0 to 54. Higher factor scores indicated more severe symptoms.

#### WISC and WAIS

In the present study, Wechsler Intelligence Scale for Children (WISC) [35] and Wechsler Adult Intelligence Scale (WAIS) [36] were employed to assess intelligence of autistic participants who aged from 12 to 16 and over 17 years old, respectively. WISC and WAIS both had verbal IQ, performance IQ and full-scale IQ. These three factors were used for correlation analysis and regression analysis.

### Data analysis

All the analyses were conducted in SPSS 24.0 software (IBM, Armonk, NY, USA). A chi-square test was employed to analyze differences among count data. Kolmogorov–Smirnov test was used to indicate whether distribution of variables was normal. Normally distributed data were presented as the mean ± standard deviation; otherwise, they were presented as the median [minimum, maximum]. For making comparisons, Mann–Whitney U test and Spearman’s rank correlation analysis were utilized as indicated. Multivariate regression analysis was conducted to explore factors associated with HRB in the autism group. Two-sided P-values < 0.05 were considered statistically significant (*P < 0.05; **P < 0.01).

## Results

### Demographic characteristics of participants in the autism and control groups

The demographic characteristics of 300 participants were shown in Table 1. Each group included 150 adolescents. The mean IQ in the autism group was 91.09 ± 4.72, while no result was provided for control group. The parental educational level, family relationships, family financial situation, and family history of psychiatric disorders are displayed in Table 1. The autism and control group significantly differed in terms of parental educational level, as well as family economic status, and family history of psychiatric disorders (P < 0.05). There were no significant differences in age, gender, residence, and family relationships between the two groups (P > 0.05).

**Table 1:** Demographic characteristics of the autism and control groups

Items	Autism group (n = 150)	Control group (n = 150)	*Z/χ2*	P
Age, median (min, max)	14.5 (12.0, 19.1)	14.5 (12.1, 19.0)	0.401	0.688
Gender			0.00	0.9999
Female	24	24		
Male	126	126		
IQ (Mean ± standard deviation)	91.09 ± 4.72	–		
Residence			1.998	0.368
City	130	137		
Town	17	10		
Rural area	3	3		
Paternal education (%)			33.48	< 0.001
Primary school	1 (0.7)	2 (1.33)		
Middle school	6 (4.0)	26 (17.33)		
High school	26 (17.3)	40 (26.67)		
Undergraduate	88 (58.7)	77 (51.33)		
Postgraduate	29 (19.3)	5 (3.34)		
Maternal education (%)			37.01	< 0.001
Primary school	1 (0.7)	6 (4.00)		
Middle school	5 (3.3)	25 (16.67)		
High school	25 (16.7)	40 (26.67)		
Undergraduate	95 (63.3)	75 (50.00)		
Postgraduate	24 (16.0)	4 (2.66)		
Family relationship (%)			2.013	0.57
Harmonious	103 (68.7)	106 (70.67)		
Fine	42 (28.0)	35 (23.33)		
Disharmony	4 (2.7)	8 (5.33)		
Parents divorced	1 (0.7)	1 (0.67)		
Financial situation (annual income CNY, %)			16.385	0.003
< 30,000	19 (12.7)	12 (8.0)		
30,000–50,000	42 (28.0)	21 (14.0)		
50,000–70,000	29 (19.3)	33 (22.0)		
70,000–100,000	31 (20.7)	30 (20.0)		
> 100,000	29 (19.3)	54 (36.0)		
Family history of mental illness (%)			5.91	0.015
Positive	14 (9.33)	4 (2.67)		
Negative	136 (88.0)	146 (97.33)		

Residence:1 = city, 2 = town, 3 = rural area

Father’s education background:1 = Primary School, 2 = Middle School, 3 = High School, 4 = Undergraduate, 5 = Postgraduate

Mother’s education background: 1 = Primary School, 2 = Middle School, 3 = High School, 4 = Undergraduate, 5 = Postgraduate

Family financial situation (annual income: yuan): 1 =  < 30,000, 2 = 30,000 ~ 50,000, 3 = 50,000 ~ 70,000, 4 = 70,000 ~ 100,000, 5 =  > 100,000

Family relationship: 1 = Harmonious, 2 = Fine, 3 = Disharmony, 4 = Parents Divorced

### Scores of AV, HCB, US, and SS subscales of AHRBI in the autism group were significantly higher than those in the control group

The results showed that the total score (Z value = − 3.47, P = 0.001), and scores of four subscales (AV, HCB, US, and SS) of AHRBI (Z value = − 4.58 ~ − 2.26, all P < 0.05) in the autism group were significantly higher than those in the control group. No significant differences were found in RB and SU subscales (Table 2). Among 38 items of the AHRBI, 21 items showed significantly higher scores (Z value = − 5.57 ~ − 2.08, all P < 0.05) in the autism group compared with those in the control group (Table S1). These results indicated that adolescents with autism had more HRB than controls, especially in AV, HCB, US, and SS subscales.

**Table 2:** Comparison of AHRBI total score and subscale scores between two groups

Total scale/Subscale	Autism group (n = 150) Median (min, max)	Control group(n = 150) Median (min, max)	Z	P
Total score	53 (38, 177)	50 (38, 88)	− 3.47	0.001
Aggression and violence	15 (10, 50)	13 (10, 29)	− 3.19	0.001
Health compromising behavior	9 (5, 21)	8 (5, 17)	− 2.26	0.024
Rule breaking	10 (7, 35)	9 (7, 18)	− 0.91	0. 363
Unprotected sex	6 (5, 25)	5 (5, 7)	− 4.58	< 0. 001
Suicide and self-injury	6 (5, 25)	5 (5, 20)	− 2.57	0.010
Substance use	6 (6, 25)	6 (6, 10)	− 1.51	0. 132

### A correlation between AHRBI scores and scores of socio-demographic data, SAS, SDS, SES, IQ, ToM, and ASSQ in the autism group

In order to explore factors potentially associated with HRB in adolescents with autism, we conducted spearman’s rank correlation analysis between AHRBI scores (AV, HCB, US, SS, and the total score of AHRBI) and the following variables: age, gender, father’s educational level, mother’s educational level, family relationship, family history of psychiatric disorders, the scores of SAS, SDS, SES, IQ, ToM, and ASSQ in the autism group. Because no significant differences were found in RB and SU subscales between two groups, we did not include these two subscales in the correlation calculation.

As shown in Table 3, AV subscale and demographic data were not correlated together. AV subscale was positively correlated with the score of SAS and SDS (r = 0.30, 0.24, respectively; all P < 0.05).

**Table 3:** Spearman’s rank correlation coefficients between the AHRBI scores and related factors

Total scale/subscale	AV	HCB	US	SS	AHRBI Total
Age	0.03	0.20^*^	0.08	0.17^*^	0.16
Father’s education background	− 0.02	− 0.05	0.12	0.04	0.02
Mother’s education background	− 0.09	− 0.19*	0.16	− 0.18*	− 0.14
Family relationship	0.16	0.16*	− 0.06	0.09	0.20*
Family Financial situation	− 0.06	− 0.02	0.02	− 0.08	− 0.02
Total score of SAS	0.30^**^	0.10	0.23^**^	0.30^**^	0.33^**^
Total score of SDS	0.24^**^	0.02	0.10	0.23^*^	0.23^**^
Total score of SES	− 0.19	− 0.14	− 0.05	− 0.26^**^	− 0.22^*^
IQ Scores					
Verbal IQ	− 0.02	− 0.17	− 0.25^*^	− 0.19	− 0.10
Performance IQ	− 0.07	− 0.13	− 0.27^*^	− 0.16	− 0.10
Full-scale IQ	− 0.04	0.03	− 0.31^*^	− 0.11	− 0.03
ToM Test					
First-Order Belief Test	− 0.05	− 0.26^**^	− 0.06	− 0.08	− 0.11
Second-Order Belief Test	− 0.02	− 0.19	− 0.23^*^	− 0.12	− 0.12
ASSQ					
Restricted and repetitive behavior	0.10	0.17	0.12	0.01	0.17
Social interaction disorder	0.07	0.17	0.04	0.04	0.13
Communication disorder	0.13	0.28^**^	0.18	0.02	0.22^*^

*P < 0.05 (2-tailed). **P < 0.01 (2-tailed)

*AV* Aggression and violence, *HCB* Health compromising behavior, *US* Unprotected sex, *SS* Suicide and self-injury, *AHRBI Total * The total score of Adolescents Health-Related Risky Behavior Inventory, *SAS* The Zung Self-rating Anxiety Scale, *SDS*  Zung Self-rating Depression Scale, *SES*  the Self-Esteem Scale, *IQ* Chinese Wechsler Intelligence, *ToM *Theory of Mind, *ASSQ* Autism Spectrum Screening Questionnaire

The score of HCB subscale was positively correlated with age, family relationship and the score of communication disorders in ASSQ (r range 0.16 ~ 0.28, P < 0.05), while negatively correlated with mother’s educational level and the score of first-order false belief task (r = − 0.19, − 0.26; P < 0.05).

No correlation was found between the score of US subscale and demographic data. The score of SAS was positively correlated with scores of US (r = 0.23, P = 0.010). The score of US subscale was markedly negatively correlated to verbal intelligence quotient, performance intelligence quotient, total intelligence scores and second-order false belief task (r = − 0.23 ~ − 0.31, all P < 0.05).

The score of SS subscale was positively correlated with age, the score of SAS, SDS (r range 0.17 ~ 0.30, P < 0.05), whereas negatively correlated with mother’s educational level and the score of SES (r = − 0.18, − 0.26, respectively; P < 0.05).

The total score of AHRBI was positively correlated with family relationship, the total scores of SAS, SDS and the score of communication disorder in ASSQ (r range 0.20 ~ 0.33, P < 0.05), while negatively correlated with SES (r = − 0.22, P = 0.025).

There was no significant correlation between HRB and gender in the autism group (We used non-parametric tests and found P value > 0.05).

### Multiple regression analysis of factors associated with HRB in adolescents with autism

Based on the results of Spearman’s rank correlation analysis, multiple linear regression was used to explore factors associated with HRB in adolescents with autism. The total score and scores of AV, HCB, US, and SS subscales of AHRBI were analyzed as dependent variables. Independent variables were chosen based on above-mentioned correlation analysis.

The results revealed that the total score of SAS was an independent risk factor for AV behavior (B = 0.27, P < 0.001); besides, communication disorder (B = 0.25, P = 0.012) and age (B = 0.24, P = 0.044) were independent factors for HCB. The total score of IQ (B = − 0.04, P = 0.043) and the score of the second-order false belief task (B = − 1.16, P = 0.033) were two independent risk factors for US. The total scores of SAS and SES were independent factors for SS. For the total score of the AHRBI, we found that total score of SAS and total score of SES were independent risk factors. The influences of the two independent variables included in the model on the total score of AHRBI were statistically significant (P < 0.05), and the specific results were summarized in Table 4.

**Table 4:** Results of multiple linear regression analysis

Model	Unstandardized coefficients		t	P	95.0% Confidence interval for B	
	B	Std. error			Lower bound	Upper bound
AV						
Total score of SAS	0.27	0.063	4.26	< 0.001	0.14	0.39
HCB						
Communication disorders	0.25	0.10	2.57	0.012	0.06	0.44
Age	0.24	0.12	2.04	0.044	0.006	0.48
US						
Total IQ Scores	− 0.04	0.02	− 2.07	0.043	− 0.08	− 0.001
Score of Second-Order False Belief Test	− 1.16	0.53	− 2.18	0.033	− 2.23	− 0.10
SS						
Total score of SAS	0.11	0.04	3.10	0.003	0.04	0.18
Total score of SES	− 0.19	0.07	− 2.60	0.011	− 0.33	− 0.04
AHRBI Total						
Total score of SAS	0.58	0.21	2.73	0.008	0.16	1.00
Total score of SES	− 1.03	0.44	− 2.32	0.023	− 1.91	− 0.15

P < 0.05 indicates significant difference

*AV* Aggression and Violence, *HCB* Health Compromising Behavior, *US* Unprotected Sex, *SS* Suicide and Self-injury, *AHRBI Total* The total score of Adolescents Health-Related Risky Behavior Inventory, *SAS* the Zung Self-rating Anxiety Scale, *SES* the Self-Esteem Scale

## Discussion

In the current study, we explored HRB and their associated factors in adolescents with autism by using AHRBI. The results showed that the total score of AHRBI and scores of AV, SS, HCB, and US subscales in the autism group were significantly higher than those in the control group, which were consistent with results of previous studies [37–40]. These results indicated that adolescents with autism were more likely to be involved in HRB.

Aggression and violence in adolescents with autism have been extensively reported in previous researches [12, 41]. Touhami et al. found that teenagers with autism were 1.4 times more likely to have AV behaviors than neurotypical teenagers [41]. Westphal et al. reported that autistic teenagers had various aggressive behaviors, including scratching, biting, and beating others [42]. Similar to these results, the current study also indicated that adolescents with autism had more aggression and violent behaviors. Based on the results of multivariate regression analysis, the present study suggested that anxiety was an independent risk factor of AV behavior in adolescents with autism. There maybe lies two reasons. On one hand, autistic individuals often suffer from emotion dysregulation, which may make it hard for them to control their impulsivity [43]. On the other hand, one of the core symptoms of autism is communication disorder, which may make it difficult for them to express their emotions clearly [44]. Based on the above two reasons, autistic individuals with anxiety may tend to rely on violence to express anger or relieve anxiety. This implied that adolescents with autism are more likely to experience aggression and violence under the influence of anxiety. Hirota et al. [13] and María et al. [45] achieved another results, in which AV behaviors in adolescents with autism were correlated to depression and family relationships, whereas these two factors were not independent predictors of AV behaviors in the regression model. This may be due to a relatively small sample size of the present research, necessitating further studies with large sample size.

In terms of HCB, the results of multivariate linear regression analysis revealed that communication disorders and age were risk factors for HCB behavior in adolescents with autism, suggesting that unhealthy eating habits of adolescents with autism were correlated to their poor language communication and increasing age, which were consistent with Geng et al.’s findings [46]. A logical explanation for this may be that it is more difficult for adolescents with communication barriers to understand why parents are more persistent in dominating a healthy diet. For example, when autistic adolescents cannot understand the health benefits of a moderate diet or eating breakfast on time, they may not develop good habits for a healthy diet but behave like overeating, excessive dieting, or skipping breakfast. As for age, we hypothesized that with increasing age in adolescence, autistic individuals may begin to pay attention to their body shape, and subsequently subjectively refrain from eating, resulting in a decrease in their food intake. It has been reported that autism often co-occurs with eating disorders [47], justifying unhealthy diets, such as excessive dieting or vomiting after overeating. An item result of HCB also showed that autistic adolescents had more physical discomforts, such as dizziness, cold sweat, and physical weakness due to excessive dieting, which were basically consistent with findings of previous studies [48, 49]. This might be related to the fact that individuals with autism often have stereotypic eating habits or comorbid with eating disorders, manifesting as less food intake which lead to physical discomforts [44, 47].

The present study confirmed that individuals with autism had more unsafe sexual behaviors than neurotypical adolescents, which was inconsistent with results of previous studies. A number of studies have reported that sexual behaviors of individuals with autism were less frequent than those of their peers or there was no significant difference between them [50–52]. We suspected that reasons for inconsistency may be caused by the following reasons. Firstly, as the results of this study showed, unprotected sex behavior was associated with IQ. We didn't limit the IQ of the individuals when they were enrolled. The IQ of the autistic group ranged from 65 to 127. Autistic adolescents with low IQ are more likely to have unprotected sex behavior. Secondly, previous studies had focused more on whether autistic adolescents had sexual behavior, while the current study aimed to indicate whether autistic adolescents could take protective measures before the occurrence of sexual behaviors, not only refer to the occurrence of sexual behavior. The above two reasons may lead to higher unprotected sexual behavior in autistic individuals than in neurotypical adolescents. The results of multivariate regression analysis showed that IQ and score of second-order false belief task were predictors of unsafe sexual behaviors in adolescents with autism, highlighting that autistic adolescents with low IQ and low ability of ToM are more likely to have unsafe sexual behavior. Opal [53] also found a significant positive correlation between sexual knowledge and IQ, that is, the lower the IQ, the worse comprehension of the sexual knowledge. Individuals with autism may not be aware of the harm that unprotected sex behavior could bring to them, such as accidental pregnancy or sexually transmitted infections. At the same time, they may not understand the measures to protect their sexual safety, either. Poor ability of second-order false belief task is also a risk factor for unprotected sexual behavior in autistic individuals. There is a primary deficit in individuals with autism, whose ToM abilities have been well established to lag behind neurotypical controls in the development of theory of mind [54, 55]. Second-order false belief task, which belongs to ToM test, refers to evaluate one's understanding of another’s belief in reality [56]. When it is difficult for autistic adolescents to understand other people's intentions or speculate on other people's thoughts, they may be easily violated by others without preparedness [57].

Rattaz et al. found that about 35.8% of autistic adolescents had self-injurious behaviors [38]. For individuals with autism, self-injurious behavior often involves cutting, inflicting burns, biting themselves, hitting a wall with their head, etc. [14]. Compared with neurotypical adolescents, the current study showed that adolescents with autism had more self-injurious behaviors and suicide attempts. Further analysis revealed that anxiety and self-esteem could predict SS in adolescents with autism. Previous studies demonstrated that self-injurious behaviors are related to anxiety, while explanation of this relationship is complicated [58, 59]. Individuals with anxiety often adopt a catastrophic cognitive emotion regulation strategy [60]. Autistic adolescents often have poor skills in emotional regulation, which may lead them to exhibit self-injurious impulsivity [61]. We hypothesized that this feature makes autistic adolescents with anxiety more prone to resort to impulsive self-injury or suicidal behavior as an escape method from distress [62]. As for low self-esteem, Titia G also found self-esteem were independently associated with severity of suicidality in autism [63]. It may increase autistic adolescents’ hopelessness, a sense of worthlessness and denial of the significance of their own existence, which may lead to ultimately self-harm and suicide [64]. This hypothesis particularly requires researchers’ attention.

There was no significant difference in the RB factor between autistic adolescents and neurotypical adolescents. The reason might be that although autistic adolescents have a risk of aggression, the impairment of social interaction and communication may disable them in finding more friends [65, 66]. Thus, they scarcely gather with others to commit illegal crimes. In addition, stereotypical features may enable them to follow rules more strictly, which may reduce the occurrence of disciplinary breaking behaviors. Besides, individuals with autism are highly under their family’s surveillance, which may lead to less disciplined violations. However, there are two items, making a significant difference between autism group and control group. One is gambling, the other is running away from home. Some researchers have found that individuals with autism may be more sensitive to numbers and have a better numerical problem-solving ability than their peers [67], leading to a better performance and a preference to certain gambling methods. In addition, autistic individuals are often accompanied by stereotyped and repetitive behaviors, which may make them more addictive [44]. These two reasons may make them more likely to indulge in gambling. As for running away from home, several potential factors may be related to this behavior (e.g., hypersensitivity to auditory or visual disturbances, such as loud noises or fluorescent lights at home, a particular interest in things outside home, or abuse at home [68, 69]). The present study did not explore potential reasons for autistic adolescents why they have gambling or leave home. Thus, further research should be carried out to eliminate this deficiency.

The current study did not find remarkable abuse of alcohol and tobacco in the autism group compared with control group, which is consistent with Mangerud et al.’s findings [70]. A previous study showed unhealthy habits in adolescents, such as smoking and drinking, were closely correlated to parents’ educational level and behaviors [71]. The majority of parents in the current study had higher educational levels, which might result in negative results. However, there were a number of items (e.g., getting drunk at a party or feeling irritation, headaches, or sleeplessness while quitting smoking) that obtained higher scores in the autism group compared with those in the control group. This maybe because adolescents with autism tend to exhibit poor self-control and repetitive and rigid behavioral patterns. Once they are exposed to alcohol, tobacco, etc., the risk of substance dependence and abuse will be far greater than neurotypical peers [72]. So attention should be paid to the prevention of alcohol and tobacco abuse in autistic adolescents.

### Limitations

The present study contains a number of limitations. Firstly, the number of subjects was relatively small. Secondly, participants in the autism group were enrolled from a single medical center, which may cause bias. Thirdly, the autistic group was mainly composed of male. Finally, the number of independent variables used in the multivariate regression analysis was limited. Therefore, further in-depth studies are warranted to eliminate the above-mentioned shortcomings, and confirm our findings.

## Conclusion

The present study showed that adolescents with autism were more likely to be involved in HRB, especially in aggression and violence, suicide or self-injury, health-compromising behavior, and unprotected sex. Different HRB have different risk factors. It is highly advised to pay more attention to HRB in adolescents with autism, so as to better understand the HRB in adolescents with autism and carry out a more comprehensive intervention for autistic adolescents.

## Supplementary Information

**Additional file 1: Table S1.** Comparison in all items of Health-related risky behaviors between autism and control group.

## Data Availability

All the clinical data used to support the findings of this study may be released upon application to the data access manager, who can be contacted at ljyuch@bjmu.edu.cn.

## Abbreviations

HRB: Health-related risky behaviors
ASD: Autism spectrum disorder
AS: Asperger's syndrome
PDD-NOS: Pervasive developmental disorder-not otherwise specified
AHRBI: Adolescent Health-Related Risky Behavior Inventory
ASSQ: Autism Spectrum Screening Questionnaire
WISC: Wechsler Intelligence Scale for Children
WAIS: Wechsler Adult Intelligence Scale
ToM: Theory of Mind Test
SAS: Zung Self-rating Anxiety Scale
SDS: Zung Self-rating Depression Scale
SES: Self-Esteem Scale
AV: Aggression and violence
SS: Suicide or self-injury
HCB: Health-compromising behavior
US: Unprotected sex
RB: Rule breaking
SU: Substance use
IQ: Intelligence quotient
YRBS: Youth Risk Behavior Survey questionnaire
ARI: Adolescent Risk Inventory
ARBS: Adolescent Risk Behavior Screening Form
RBQ-A: Adolescent Risk Behavior Questionnaire
CARS: Childhood Autism Rating Scale
K-SADS-PL: Schedule for Affective Disorders and Schizophrenia for School-Age Child-Present and Lifetime Version
