# Supplementary material for: Health-related risky behaviors in Chinese adolescents with autism: a cross-sectional study
Source: Child Adolesc Psychiatry Ment Health. 2021 Jul 30;15:39. doi: 10.1186/s13034-021-00390-6 (PMC8325271; doi:10.1186/s13034-021-00390-6)
Supplement: Supplementary file 1 — Additional file 1: Table S1. Comparison in all items of Health-related risky behaviors between autism and control group. [file 13034_2021_390_MOESM1_ESM.doc]

Table S1

| Items | Autism group  (n=150)  Median score  (min, max) | Normal group  (n=150)  Median score  (min, max) | Z | P |
| --- | --- | --- | --- | --- |
| **Aggression and violence behaviors** |  |  |  |  |
| Have you been involved in a physical fight? | 1(1,5) | 1(1,3) | -5.57 | <0.001 |
| Have you driven (a bicycle, scooter, or car) without considering consequences (e.g., at high speed under the influence of certain substances) | 1(1,5) | 1(1,3) | -4.31 | <0.001 |
| Have you intentionally hit, shoved, kicked or confined someone? | 1(1,5) | 1(1,3) | -3.54 | <0.001 |
| Have you blackmailed others for money? | 1(1,5) | 1(1,3) | -3.23 | 0.001 |
| Have you ever carried a weapon (e.g., a gun, dagger, stick, etc.)? | 1(1,5) | 1(1,5) | -2.72 | 0.006 |
| Have you bullied, threatened or intimidated someone? | 1(1,5) | 1(1,5) | -2.09 | 0.036 |
| Have you gotten revenge on someone? | 1(1,5) | 1(1,5) | -2.08 | 0.037 |
| Have you made fun of others for their physical defects or appearance? | 1(1,5) | 1(1,4) | -1.37 | 0.17 |
| Have you verbally attacked someone? | 2(1,5) | 2(1,5) | -0.98 | 0.327 |
| Have you destroyed others’ properties? | 2(1,5) | 2(1,5) | -0.61 | 0.545 |
| **Health compromising behaviors** |  |  |  |  |
| Have you ever had physical discomforts such as dizziness, sweating, and faintness due to excessive dieting? | 1(1,5) | 1(1,3) | -4.52 | <0.001 |
| Have you ever eaten too much or vomited after overeating? | 1(1,5) | 1(1,4) | -1.47 | 0.142 |
| How often do you drink milk/soymilk? | 1(1,5) | 3(1,5) | -1.28 | 0.2 |
| Don't you take part in any kind of sports? | 2(1,5) | 2(1,5) | -1.00 | 0.319 |
| How often do you have breakfast? | 1(1,5) | 1(1,5) | -0.08 | 0.941 |
| **Rule breaking behaviors** |  |  |  |  |
| Have you gambled ? | 1(1,5) | 1(1,1) | -3.37 | 0.001 |
| Have you ever run away from home ? | 1(1,5) | 1(1,5) | -2.08 | 0.037 |
| Have you cheated in tests ? | 1(1,5) | 1(1,5) | -1.29 | 0.197 |
| Have you skipped classes or played truant from school ? | 1(1,5) | 1(1,5) | -0.90 | 0.368 |
| Steal money | 1(1,5) | 1(1,3) | -0.44 | 0.657 |
| Have you lied to his/her family members (such as grandparents, parents, brothers and sisters)? | 2(1,5) | 2(1,5) | -0.21 | 0.836 |
| Have you ever been warned, demerit recorded, punished or dropped out of school due to your disobedience ? | 1(1,5) | 1(1,5) | -0.86 | 0.931 |
| **Unprotected sex behaviors** |  |  |  |  |
| Have sexual intercourse with strangers | 1(1,5) | 1(1,1) | -3.97 | <0.001 |
| Have sexual intercourse with over two people at the same time | 1(1,5) | 1(1,1) | -3.68 | <0.001 |
| Do you use contraception when having sexual intercourse? | 1(1,5) | 1(1,2) | -3.44 | 0.001 |
| Do you drink alcohol or use drugs before having sexual intercourse? | 1(1,5) | 1(1,1) | -2.86 | 0.004 |
| Boy: Have you ever caused a girl pregnant? Girl: have you ever been pregnant accidentally? | 1(1,5) | 1(1,1) | -2.47 | 0.014 |
| **Suicide and self-injury behaviors** |  |  |  |  |
| Do you have any ideas of suicide? | 1(1,5) | 1(1,5) | -2.92 | 0.004 |
| Do you have any plan to commit suicide? | 1(1,5) | 1(1,3) | -2.68 | 0.007 |
| Have you tried to cut or burn yourself? | 1(1,5) | 1(1,4) | -2.43 | 0.015 |
| Have you ever committed suicide? | 1(1,5) | 1(1,4) | -1.58 | 0.115 |
| Have you ever intentionally hurt yourself by biting, scratching, hitting, etc.? | 1(1,5) | 1(1,5) | -1.54 | 0.123 |
| **Substance use behaviors** |  |  |  |  |
| Have you drunk alcohol out of control at a party? | 1(1,5) | 1(1,1) | -3.37 | 0.001 |
| Have you been irritable, headache, or sleepless while quitting smoking? | 1(1,4) | 1(1,4) | -2.85 | 0.004 |
| Have you drunk alcohol to save face? | 1(1,5) | 1(1,3) | -2.09 | 0.036 |
| Have you been drunk? | 1(1,5) | 1(1,2) | -1.69 | 0.09 |
| Have you smoked under the pressure of companion? | 1(1,3) | 1(1,3) | -1.42 | 0.155 |
| How often do you smoke? | 1(1,4) | 1(1,3) | -1.27 | 0.204 |
